# Long-Term Urban Air Pollution Drives Multi-Stage Neuropsychiatric Disorder Trajectories: A Prospective Cohort Study

**DOI:** 10.3390/toxics14010004

**Published:** 2025-12-19

**Authors:** Yuanyuan Song, Shiqing Zhang, Siru Yang, Xiaoke Gao, Lei Shi, Jinjian Chen, Kaili Lin, Jun Yang

**Affiliations:** 1 School of Public Health, Guangzhou Medical University, Guangzhou 511436, China; 2023210506@stu.gzhmu.edu.cn (Y.S.); gzyangsiru@126.com (S.Y.); 15884862506@163.com (X.G.); chenjinjian@gzhmu.edu.cn (J.C.); 2 State Key Laboratory of Bioactive Molecules and Druggability Assessment, Guangdong Basic Research Center of Excellence for Natural Bioactive Molecules and Discovery of Innovative Drugs, Jinan University, Guangzhou 510632, China; sqzhang@jnu.edu.cn (S.Z.); t_shilei@jnu.edu.cn (L.S.); 3 JNU-HKUST Joint Laboratory for Neuroscience and Innovative Drug Research, College of Pharmacy, Jinan University, Guangzhou 510632, China; 4 Guangdong Province Key Laboratory of Pharmacodymamic Constituents of TCM & New Drugs Research, Guangdong Hong Kong-Macau Joint Laboratory for Pharmacodynamic Constituents of TCM and New Drugs Research, Jinan University, Guangzhou 510632, China

**Keywords:** air pollution, neuropsychiatric disorders, multi-state model, progression trajectory

## Abstract

Neuropsychiatric disorders constitute an escalating public health challenge worldwide, with growing evidence suggesting that environmental factors like air pollution may contribute substantially. This prospective cohort study investigated the associations between long-term exposure to fine particulate matter (PM_2.5_) and nitrogen oxides (NO_x_) and the progression of eight neuropsychiatric disorders among 502,356 UK Biobank participants. Using multi-state models, we analyzed three distinct trajectory stages: stage 1 (transition from baseline healthy status to PHQ-4-positive mood disorders), stage 2 (transition from baseline to ICD-10-diagnosed disorders), and stage 3 (progression from PHQ-4-positive status to clinical diagnosis). Nonlinear exposure–response relationships were subsequently characterized using restricted cubic spline (RCS) regression models. The findings indicated that exposure to both PM_2.5_ and NO_x_ per IQR increase was strongly associated with stage 1, with a corresponding hazard ratio of 1.28 (95% CI: 1.27–1.30) and 1.10 (95% CI: 1.09–1.11). Across the three stages, the risk pattern evolved from being broadly significant to one characterized by disease-specific significance. Alzheimer’s disease was consistently identified as the condition with the strongest association and highest risk linked to air pollution. Specifically, hazard ratios across stages were as follows: 1.08–1.13 in stage 2 and 1.14–1.20 in stage 3 for PM_2.5_; and 1.04–1.05 in stage 2 and 1.05–1.10 in stage 3 for NO_x_. Subgroup analyses identified heightened vulnerability in females (particularly subjects with depression, Parkinson’s disease, and sleep disorders), younger individuals, and socioeconomically deprived populations. These findings underscore the importance of considering air pollution as a modifiable risk factor in the prevention of neuropsychiatric disorders.

## 1. Introduction

Neuropsychiatric disorders represent a critical global health crisis, encompassing a spectrum of mental illnesses (e.g., major depressive disorder, bipolar disorder, anxiety disorder, and psychotic disorders) and neurological diseases (e.g., Alzheimer’s disease, Parkinson’s disease, epilepsy, and stroke) [1]. These disorders impose a substantial burden of chronic disability, especially as populations age and environmental pressures intensify [2,3]. Approximately one billion individuals worldwide are affected by mental disorders, with depression and anxiety being the most prevalent [4]. Stroke affects approximately 12.2 million persons yearly, while Alzheimer’s disease and related dementias currently afflict 55 million people, with a projected tripling of cases by 2050 due to population aging and environmental factors [5,6]. Consequently, early identification of environmental risk factors and prodromal symptoms of neuropsychiatric disorders has emerged as a critical priority for intervention.

Emerging evidence implicates environmental exposures, particularly air pollution, as modifiable risk factors in neuropsychiatric pathogenesis. Air pollution exposure is widely regarded as a leading environmental risk factor for various diseases and premature mortality [7], primarily due to its harmful effects on the respiratory and cardiovascular systems [8,9]. Previous studies suggest that long-term exposure to PM_2.5_ and NO_x_ not only causes damage to the respiratory and cardiovascular systems [10] but may also adversely affect the central nervous system. Proposed mechanisms include pollutant-induced neuroinflammation and oxidative stress, which in turn contribute to cognitive impairment and greater susceptibility to mental illness [11]. Elevated PM_2.5_ and NO_x_ exposure significantly increases the risk for Alzheimer’s disease [12], schizophrenia [13], major depressive disorder [14,15], dementia [15], and Parkinson’s disease [16]. Despite established associations with respiratory and cardiovascular morbidity, the neurobiological consequences of long-term air pollution exposure remain underexplored, particularly its dose-dependent effects on central nervous system vulnerability across disease trajectories. Most studies have focused on acute neurological outcomes, leaving significant gaps in understanding how prolonged exposure shapes the progression from subclinical prodromes to clinical disorders.

The prodromal phase, characterized by emergent signs and symptoms preceding formal diagnostic thresholds, represents a crucial window for the early detection and intervention of diseases [17]. In-depth investigation of this early stage is essential for elucidating pathogenic mechanisms, identifying predictive biomarkers, enabling timely strategies to mitigate disease progression and burden, and ultimately leading to more effective prevention and treatment of neuropsychiatric disorders. Notably, emotional disturbances, particularly depression and anxiety states, are frequently prominent prodromal features across many neuropsychiatric disorders [18], and similar prodromal phases were observed in patients with dementia, psychosis, and bipolar disorder [19,20].

Therefore, leveraging the UK Biobank cohort, this longitudinal study aims to elucidate the effects of prolonged exposure to urban air pollutants (PM_2.5_ and NO_x_) on the developmental trajectories of prevalent neuropsychiatric disorders, spanning from prodromal stages to clinical onset. Employing multi-state models and RCS analyses, the study comprehensively assesses the relationships between long-term exposure to PM_2.5_ and NO_x_ and the development of Alzheimer’s disease, anxiety disorder, stroke, depression, epilepsy, migraine, Parkinson’s disease, and sleep disorders. The present longitudinal study seeks to provide critical evidence for environmental risk reduction and prevention strategies within urban health policy frameworks.

## 2. Methods

### 2.1. Study Design and Population

**Figure 1:**
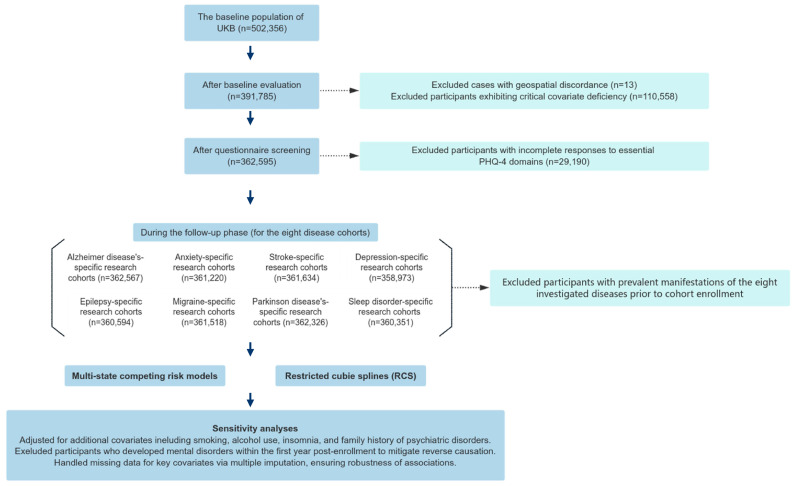
The design and workflow of this study. Excluded participants with a history of the mental illness at baseline: Alzheimer’s disease (*n* = 28), anxiety disorder (*n* = 1375), stroke (*n* = 961), depression (*n =* 3622), epilepsy (*n =* 2001), migraine (*n =* 1077), Parkinson’s disease (*n =* 269), and sleep disorders (*n =* 2244).

Leveraging the rich resources of the UK Biobank (Application Number 95736; http://www.ukbiobank.ac.uk/ (accessed on 25th October 2023)), a large-scale prospective cohort of 502,536 individuals across England, Scotland, and Wales in the UK was selected that contained data on genetics, medical imaging, and environmental exposures. The population of the UK increased from approximately 60.8 million in 2006 to 62.8 million in 2010. From the initial 502,536 participants (aged 32–88 years) recruited during 2006–2010, we excluded individuals with missing geocoded residential addresses (*n* = 13), incomplete core covariates (*n* = 110,558), or failure to respond to baseline questionnaires (*n* = 29,190). Following the additional exclusion of prevalent cases of target diseases (Figure 1), the final analytical cohort comprised incident disease-free participants for longitudinal investigation. The UK Biobank has secured ethical approval from the National Research Ethics Service, and all participants have given their informed written consent [21].

### 2.2. Outcome Assessment

This study employed the Patient Health Questionnaire-4 (PHQ-4) scale from the UK Biobank, which includes four items: “Frequency of depressed mood over the past two weeks”, “Frequency of lack of enthusiasm or interest”, “Frequency of nervousness or anxiety”, and “Frequency of fatigue or drowsiness”. Participants scoring ≥ 6 (on a 0–12 scale) were classified as PHQ-4 positive for mood disorders [22]. Disease endpoints were extracted from hospitalization records via the primary and secondary diagnosis fields and employing ICD-10 (WHO International Statistical Classification of Diseases and Related Health Problems, 10th Revision) classification protocols. Relevant ICD-10 codes included depressive disorders (F32–F33), anxiety disorder (F40–F41), Alzheimer’s disease (G30, F00), Parkinson’s disease (G20), epilepsy (G40–G41), migraine (G43), sleep disorders (G47), and stroke (I63–I64). The follow-up spanned from the baseline assessment during 2006–2010 to 30 October 2022.

### 2.3. Air Pollution

Annual PM_2.5_ and NO_x_ pollution data were obtained from the UK’s Department for Environment, Food and Rural Affairs (DEFRA) platform (https://uk-air.defra.gov.uk/data/pcm-data (accessed on 28 October 2023), which provides 1 km × 1 km, near-surface air pollutant estimates across the UK from 2002 to 2022. These data were derived from the Pollution Climate Mapping (PCM) model coupled with the Atmospheric Dispersion Modeling System (ADMS) [23], demonstrating moderate correlation with the observed data from air pollution stations (R^2^ > 0.5) [24,25]. Using each participant’s geocoded residential coordinates, annual average PM_2.5_ concentrations were assigned via an inverse-distance-weighted method and grid-based linear interpolation from monitoring stations. Residential duration at each address (including relocations) was then calculated in cumulative days, enabling us to derive personalized, state-specific, annualized exposure estimates for both PM_2.5_ and NO_x_ within a multi-state modeling framework.

### 2.4. Measurement of Covariates

To mitigate confounding, we adjusted for baseline covariates encompassing demographic, regional, socioeconomic, and lifestyle factors. Specifically, a series of covariates were selected based on prior studies and the focus of our research, including age (years, continuous); gender (male or female); body mass index (BMI; underweight, normal weight, overweight, or obesity); ethnicity (dichotomized as White and non-White due to small sample sizes of other ethnic groups); Townsend deprivation index (TDI; as a three-level variable based on tertiles); employment status (employed, retired, or other); education level (dichotomized at 20 years); physical activity level (assessed by the International Physical Activity Questionnaire, IPAQ; high, moderate, or low); smoking status (current, former, or non-smoker); alcohol consumption (current, former, or non-drinker); insomnia status (current, former, or no insomnia); and family history of depression or Parkinson’s disease. Detailed information is shown in Table S1. By controlling for these covariates, the study effectively minimized the biases caused by confounding factors, enhancing the scientific rigor and accuracy of the results.

### 2.5. Statistical Analysis

A phased analysis method was used to assess the effects of air pollution exposure on the progression of neuropsychiatric diseases. Initially, descriptive statistics were applied to systematically evaluate the baseline characteristics of the participants: continuous variables were expressed as mean ± standard deviation (SD) and categorical variables as frequency and proportions. Next, we utilized a multi-state competing risk model (MSM) to analyze the progression of neuropsychiatric disorders [26]. Built upon the Cox proportional hazards framework, this model facilitated our evaluation of the influence of air pollution exposure on transitions across three distinct stages [27,28]. The stages we defined were as follows: Baseline (excluding the population already diagnosed with the eight diseases under study) to the diagnosis of mood disorders (PHQ-4 score ≥ 6) as stage 1; (2) baseline to the onset of neuropsychiatric disorders (ICD-10 diagnosis) as stage 2; (3) from diagnosis of mood disorders to the onset of neuropsychiatric disorders as stage 3. To account for potential confounders, the model adjusted for a range of covariates, including demographic factors (age, gender, and race), socioeconomic status (income, education level, and occupation), health-related variables (body mass index and physical activity), and personal behaviors (smoking, alcohol consumption, and insomnia). In our subgroup analyses, we performed stratified analyses based on age groups, gender, and economic status, using interaction terms to evaluate the differences in risk among the various subgroups. Furthermore, to investigate disease-specific progression pathways and avoid potential confounding across different disorders, we constructed independent multi-state models for each of the eight neuropsychiatric diseases. To delve deeper into the intricate relationship between pollutant exposure and disease outcomes, we utilized a restricted cubic spline model with three knots of freedom to examine the nonlinear exposure–response relationship [29].

### 2.6. Sensitivity Analyses

To assess the stability of the model, we performed multiple sensitivity analyses. Firstly, we included additional health-related covariates, including personal diagnoses of hypertension, diabetes, stomach ulcers, and a family history of neuropsychiatric disorders. Secondly, to account for potential pre-enrollment exposures, participants who developed mental disorders within the first year of enrollment were excluded. Furthermore, we applied multiple imputations using chained equations to fill in missing data for several important covariates.

All statistical analyses were conducted using R version 4.3.1, and the primary models were implemented with the “mstate” package. Statistical significance was defined as a two-tailed *p*-value less than 0.05.

## 3. Results

### 3.1. Study Population and Air Pollution Exposure

**Figure 2:**
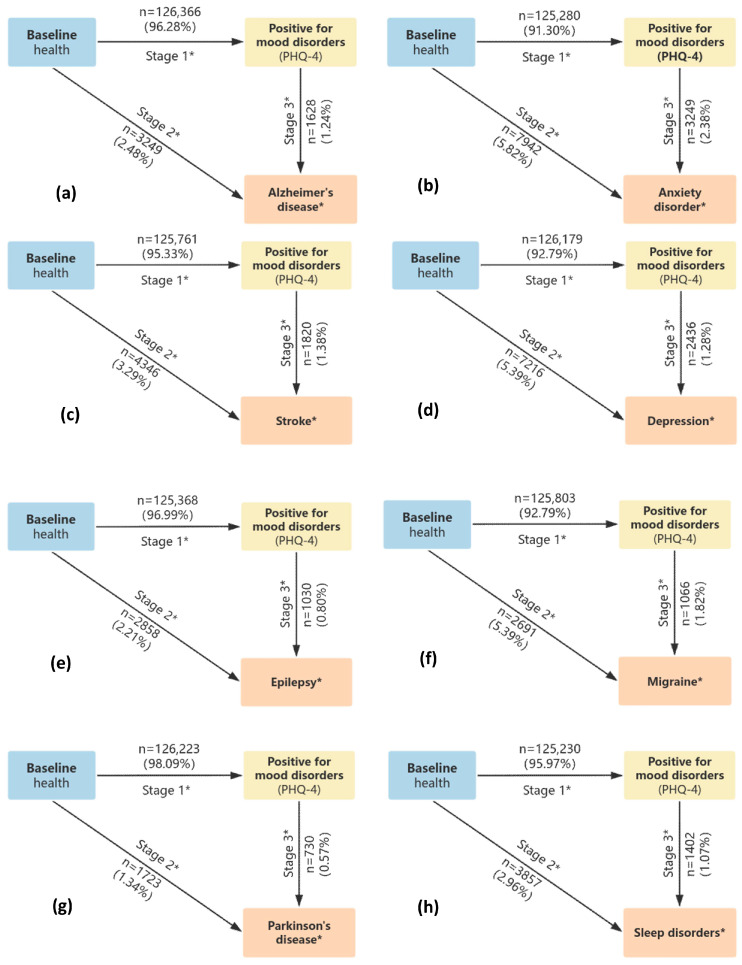
Number (percentage) of participants from baseline to emotional disorder positivity and subsequent neuropsychiatric diseases. Note: * This study modeled a three-stage progression of disease: Stage 1 was defined as from the baseline to the diagnosis of mood disorders but before the onset of neuropsychiatric disorders. Stage 2 was defined as the disease progression from baseline to an ICD-10 clinical diagnosis and stage 3 as from a PHQ-4-positive status to a subsequent clinical diagnosis. Each disease is designated as an absorbed state. Once entered, transitions to stage 1 and stage 2 will no longer occur. (**a**–**h**) represent the count and percentage of participants for each disease.

The study enrolled 502,356 participants (mean age: 60.99 years) for questionnaire-based analysis. Over a median follow-up duration of 17.7 years, females constituted 54.4% of the cohort (Table 1). Of the initial cohort, 1297 participants were lost to follow-up and 44,499 died during the study period. Questionnaire-based screening identified 162,160 participants with potential emotional disorders. Ultimately, 47,243 incident cases of neuropsychiatric disorders were confirmed at the endpoint, with the following distribution: Alzheimer’s disease (4877), anxiety disorder (11,191), stroke (6166), depression (9652), epilepsy (3888), migraine (3757), Parkinson’s disease (2453), and sleep disorders (5259). The progression of participant numbers for each disease from baseline to the final state is detailed in Figure 2a–h. During the follow-up period, the mean annual air pollutant concentrations of PM_2.5_ and NO_x_ reached 9.99 μg/m^3^ and 18.19 μg/m^3^, respectively (Table S2).

### 3.2. Main Results of the Multi-State Model

In the multi-state model analysis, long-term exposure to PM_2.5_ and NO_x_ was found to significantly increase the risk of developing several psychiatric disorders, with PM_2.5_ demonstrating a stronger effect than NO_x_. Specifically, in stage 1 (defined as from baseline to the diagnosis of mood disorders but before the onset of neuropsychiatric disorders), the risk estimate associated with per IQR increase in PM_2.5_ [hazard ratio (HR) = 1.28; 95% CI: 1.27, 1.30] was higher than NO_x_ (HR = 1.10; 95% CI: 1.09, 1.11). Upon entering stage 2 (Table 2), risk estimates began to diverge markedly among different neuropsychiatric diseases. For PM_2.5_, HRs were between 1.06 and 1.13, with significant risks remaining for Alzheimer’s disease (HR = 1.13, 95% CI: 1.06, 1.21) and anxiety disorder (HR = 1.10; 95% CI: 1.06, 1.32). In contrast, effect estimates for NO_x_ mostly decreased to approximately 1.05. Specifically, higher NO_x_ exposure was associated with an increased risk for disease progression in stage 2 for patients with anxiety (HR = 1.05; 95% CI: 1.02, 1.09) or depression (HR = 1.05, 95% CI: 1.02, 1.09). In stage 3, the long-term cumulative effect of PM_2.5_ was most pronounced, with HRs generally ranging from 1.05 to 1.10—consistently higher than those for NO_x_ (around 1.07). Alzheimer’s disease exhibited the highest risk among all conditions in this stage for PM_2.5_ (HR = 1.20; 95% CI: 1.09, 1.32), indicating the strongest association, followed by depression (HR = 1.17; 95% CI: 1.10, 1.24), stroke (HR = 1.17; 95% CI: 1.08, 1.27), and migraine (HR = 1.10; 95% CI: 1.01, 1.19).

### 3.3. Subgroup Analysis and Exposure–Response Associations

**Figure 3:**
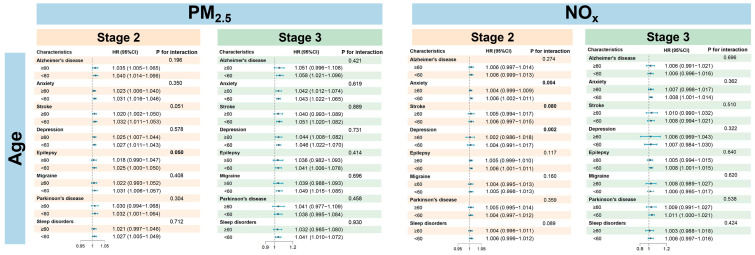
Relationships of PM_2.5_ and NO_x_ exposure and disease progression at stage 2 and stage 3, stratified by age. Stage 2 was defined as the disease progression from baseline to an ICD-10 clinical diagnosis and stage 3 as from a PHQ-4-positive status to a subsequent clinical diagnosis.

**Figure 4:**
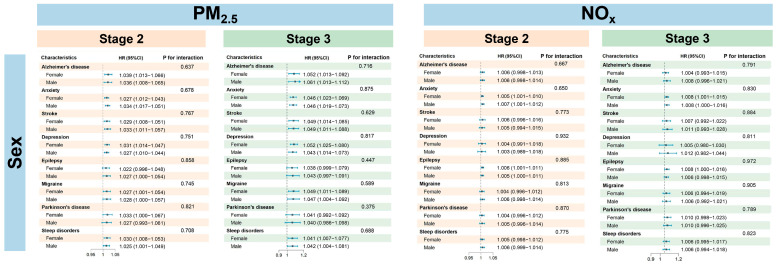
Relationships of PM_2.5_ and NO_x_ exposure and disease progression at stage 2 and stage 3, stratified by sex. Stage 2 was defined as the disease progression from baseline to an ICD-10 clinical diagnosis and stage 3 as from a PHQ-4-positive status to a subsequent clinical diagnosis.

**Figure 5:**
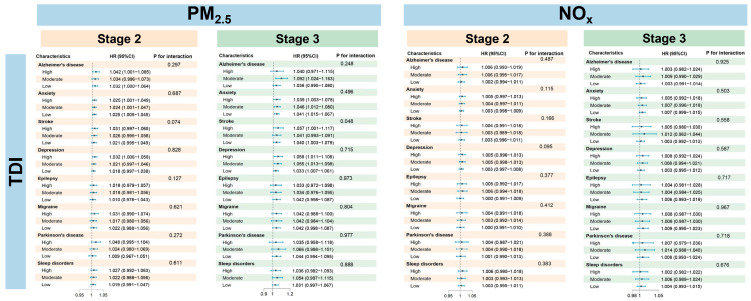
Relationships of PM_2.5_ and NO_x_ exposure and disease progression at stage 2 and stage 3, stratified by Townsend index. Stage 2 was defined as the disease progression from baseline to an ICD-10 clinical diagnosis and stage 3 as from a PHQ-4-positive status to a subsequent clinical diagnosis.

Stratified analyses revealed distinct patterns of vulnerability to PM_2.5_ and NO_x_ exposure based on sex, age, and TDI (Figure 3, Figure 4 and Figure 5). In stage 1, the subgroup analysis revealed no significant heterogeneity in gender and age groups. For PM_2.5_, the majority of outcomes in stage 2 (such as anxiety and stroke) indicated a higher risk for males compared to females, while conditions like depression, Parkinson’s disease, and sleep disorders showed greater susceptibility in females. In stage 3, the risk for depression continued to be elevated in females, whereas gender differences for other outcomes diminished. The overall association with NO_x_ was not significant, except for stroke in stage 3, where males exhibited a significantly higher risk than females. With respect to age, younger individuals were more vulnerable to air pollution, showing heightened risks across all stages of disease progression. Similarly, individuals with lower socioeconomic status exhibited a consistently elevated risk, particularly in stage 1 (Table S3), with HRs as follows: PM_2.5_ (HR = 1.082; 95% CI: 1.077, 1.087) and NO_x_ (HR = 1.011; 95% CI: 1.009, 1.012). Significant effect modification by age was observed primarily in stage 2. For PM_2.5_, a interaction with age was found for stroke (*p* = 0.051) and epilepsy (*p* = 0.050). For NO_x_, significant interactions with age were identified for anxiety (*p* = 0.004), stroke (*p* = 0.002), and depression (*p* = 0.002), indicating that age is a key moderating factor for the impact of NO_x_ on these diseases. Furthermore, gender and TDI did not show significant effects in the interactions of either pollutant, and no significant interactions were observed in stage 3, suggesting that the interaction effects are primarily concentrated in specific stages.

**Figure 6:**
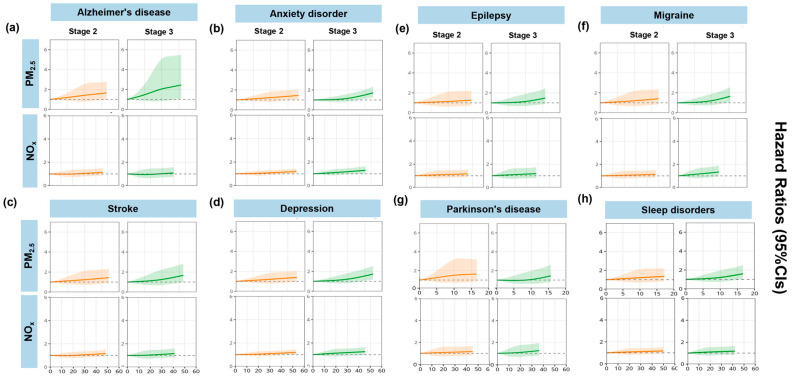
Exposure–response relationships of air pollution exposure and disease progression at stage 2 and stage 3. Note: Stage 2 was defined as the disease progression from baseline to an ICD-10 clinical diagnosis and stage 3 as from a PHQ-4-positive status to a subsequent clinical diagnosis. Letters (**a**–**h**) denote the individual analysis panels for the eight diseases.

Figure 6 presents the exposure–response relationships between air pollutants and risks of psychiatric disorders. We observed a general upward trend in the exposure–response curves, suggesting that as pollutant concentrations increased, the risk of psychiatric disorders also increased. Across different stages, the exposure–response relationship was not simply linear but varied across stages. In stage 1, the RCS curve exhibited a “U” shape with a turning point, whereas in other stages, it demonstrated a monotonic increasing trend with rising pollutant exposure.

### 3.4. Sensitivity Analyses

Sensitivity analyses shown in Tables S4–S6 revealed that incorporating extra covariates and removing participants who developed the disease within the first year of enrollment did not materially alter the results. To ensure the model’s robustness, missing data for several key covariates were addressed through multiple imputation using chained equations. These assessments collectively indicated that the findings had strong generalizability and offered a dependable foundation for studies in environmental epidemiology.

## 4. Discussion

This longitudinal multi-stage analysis advances understanding of air pollution’s neuropsychiatric impacts by mapping exposure–response relationships across the full disease continuum from the initial onset of psychiatric disorders to the diagnosis of neuropsychiatric disorders. The findings demonstrated that sustained exposure to PM_2.5_ and NO_x_ had differential effects across the stages of neuropsychiatric disorders. Notably, the early stages of psychiatric disorders exhibited increased sensitivity to these pollutants, a pattern validated through stratified analyses that revealed sex-specific and socioeconomic vulnerabilities. Specifically, long-term exposure to PM_2.5_ was linked to an increased overall risk of developing mental illnesses compared to exposure to NO_x_.

The present study demonstrates that long-term air pollution exposure is a significant contributor to the risk of various neurological and psychiatric disorders. This evidence is corroborated by an earlier review [13], which likewise identified associations between air pollution and increased incidence risks for depression, anxiety, bipolar disorder, and psychosis and suicide risk and reported that for every 10 μg/m^3^ increase in PM_2.5_ concentration, the odds ratio (OR) for depression was 1.102 (95% CI: 1.023–1.189) per 10 μg/m^3^ increase in PM_2.5_ concentration [30]. Additionally, a systematic review on Alzheimer’s disease found that five air pollutants were associated with an overall OR of 1.32 (95% CI: 1.09–1.61) [31]. Moreover, NO_2_ was associated with the risk of depression by 0.61% (95% CI: 0.31–0.92%) among individuals aged over 64 [32]. Another study demonstrated that NO_2_, NO_x_, and PM_2.5_ are associated with cognitive impairment and identified NO_2_ as the primary driver of overall cognitive decline [33]. Similarly, research based on the UK Biobank database found that PM_2.5_ exposure was associated with a 196% increase in anxiety risk, while NO_2_ exposure increased schizophrenia risk by 95% and bipolar disorder risk by 43% [13]. Meanwhile, discrepant studies reported that PM_2.5_ exposure was not associated with mental or behavioral disorders or suicide mortality, while SO_2_ exposure was significantly associated with suicide mortality [34]. A Taiwan Biobank-based study indicated no significant relationship between PM_2.5_ and affective psychiatric disorders [35]. Such discrepancies may stem from variations in regional databases, study populations, and outcome variable definitions among the studies. Notably, our multi-state modeling approach, which captures temporal disease progression rather than cross-sectional associations, may also explain the divergent findings compared to traditional case–control designs.

The stage-specific pollutant effects observed in this study showed that the harmful effects of different pollutants varied across stages of disease progression. PM_2.5_ and NO_x_ presented significant risks during the initial phase of disease progression, particularly from health to mental disorders, consistent with several multi-state study conclusions. A U.S. study on Alzheimer’s disease progression reported a high transition probability in the early stage (mild cognitive impairment to mild Alzheimer’s disease), with rates of 12.8% at one year and 29.8% at ten years [36]. Regarding the dynamic progression of type 2 diabetes, exposure to PM_2.5_, PM_10_, NO_x_, and NO_2_ was strongly predictive of the healthy-to-diabetes transition, with an HR per IQR increase of 1.63 (95% CI: 1.59–1.67), 1.66 (95% CI: 1.62–1.69), 1.39 (95% CI: 1.37–1.42), and 1.49 (95% CI: 1.46–1.51), respectively [37]. Similarly, another multi-state study also showed that increases in PM_2.5_ and NO_2_ were linked to a 3% (95% CI: 2–5%) increased risk of progressing from health to the first stage of cardiometabolic disease (FCMD) [38]. When it came to the transition from mood disorders to neuropsychiatric disorder onset, PM_2.5_ was associated with elevated risk for most neuropsychiatric disorders. NO_x_ also showed increased risk from baseline to the onset of anxiety and depression. However, no significant associations were found for other neuropsychiatric disorders, possibly due to weaker links between long-term pollutant exposure and severe psychiatric illnesses or the limited number of cases in these categories. Furthermore, a multi-stage study on bipolar disorder in South Korea showed that disease progression was strongly associated with psychiatric comorbidities and age at onset [39]. These results imply that air pollution exerts widespread effects across the disease spectrum, affecting disease occurrence, progression, and mortality risk from health to multiple disorders.

Emerging evidence from toxicology and neurobiology provides a mechanistic framework for understanding the associations between air pollution exposure and neuropsychiatric disease progression. Chronic exposure to PM_2.5_ and NO_x_ likely exerts neurotoxic effects through multiple intersecting pathways, with neuroinflammation emerging as a central pathogenic mechanism [40]. This chronic inflammatory state is demonstrably linked to progressive cognitive and behavioral deficits in animal models [41]. Additionally, nitrogen oxides display a concentration-dependent duality; while nitric oxide (NO) has neuroprotective effects at physiological levels, it can become neurotoxic at the higher concentrations commonly found in polluted environments [42]. Mechanistic studies, particularly in migraine models, implicate pollutant-induced oxidative stress (e.g., reactive oxygen species bursts) and subsequent activation of downstream processes to specific neurological outcomes [43]. Such mechanisms may explain the selective association between NO_x_ exposure and anxiety/depression progression observed in our cohort.

Our stratified analyses revealed significant heterogeneity in air pollution-related risks across demographic subgroups, underscoring the complex interplay between environmental exposures, biological sex, age, and socioeconomic status. Females demonstrated greater susceptibility to prodromal mood disorder progression (stage 1) compared to males [44], whereas males demonstrated relatively elevated risks during later disease progression stages (stages 2 and 3). This observation aligns with epidemiological evidence indicating that females generally experience higher rates of depression and anxiety, often with greater severity and comorbidity [45,46]. However, for certain neuropsychiatric disorders such as stroke, epilepsy, and migraine, this pattern is reversed under the female protective model [47]. Potential biological mediators for these sex-specific differences include the influence of sex hormones and epigenetic modifications such as DNA methylation [48].

For age, our results diverged from previous studies. We found that younger individuals were more susceptible during psychiatric disease progression, whereas other studies typically reported higher incidence rates among older adults [49], especially those aged 60 to over 80, with a rapidly increasing disease burden. There are several potential reasons for this discrepancy. This difference may be related to the mood disorder indicators used in our study, which are more prevalent in younger populations. Numerous studies have established that the onset of most psychiatric disorders peaks during adolescence [50], while the elderly peak for mood disorders occurs at 75 years and above [51]. Our cohort mainly comprised individuals aged approximately 50 to 60, which may account for the observed discrepancies. Furthermore, evidence suggests a trend toward earlier onset of psychiatric disorders, with declining age-standardized prevalence rates and higher risks among younger populations for most conditions [49,52]. Socioeconomic status (SES) emerged as a dominant modifier, with low-SES individuals facing higher progression risks across all stages of psychological and cognitive disease development [53]. Earlier research also indicated that low-SES individuals experienced higher rates of severe and chronic mental health disorders [54], and a Mendelian randomization analysis revealed bidirectional causal relationships between poverty and multiple psychiatric conditions [22]. The living and commuting patterns of low-SES individuals may have greater exposure to pollutants, emphasizing the vital role of economic factors in the progression of neuropsychiatric diseases and the effectiveness of interventions [55]. This investigation presents several strengths that enhance the robustness of its findings. First, the prospective cohort design and large sample size improve statistical power compared to cross-sectional studies. Second, unlike conventional single-stage analyses, the multi-state modeling framework enabled characterization of air pollution effects across distinct disease transitions from asymptomatic states through subthreshold mood disturbances to diagnosed psychiatric/neurological disorders. Third, the use of the PHQ-4 questionnaire complemented traditional diagnostic codes to assess positive mood disorder symptoms, effectively identifying early vulnerable populations and providing precise information for early prevention and intervention [56]. Finally, the comprehensive neuropsychiatric battery in this study provides broader insights than disease-specific air pollution research. Despite these strengths, several limitations warrant consideration. First, pollutant exposure was assessed solely based on residential addresses, without accounting for exposure with non-homebound lifestyles or occupational pollutant exposures. Second, although associations between pollutants and psychiatric disorders were identified, causal relationships were not explored. Future research should adopt more complex designs to investigate the causal mechanisms of pollutant exposure on mental health. Moreover, the sample primarily comprised middle-aged and elderly individuals from relatively affluent regions. Generalization to younger populations or socioeconomically disadvantaged areas requires caution due to potential differences in environmental exposures and health determinants. Future studies should conduct broader cross-regional and cross-population research to enhance the universality of conclusions. Finally, this study did not classify existing diseases by severity nor construct more complex multi-state models, which represent important directions for future research.

## 5. Conclusions

This longitudinal analysis demonstrates that long-term exposure to ambient PM_2.5_ and NO_x_ accelerates neuropsychiatric disease progression across urban populations, with particularly pronounced effects during early stages of disease development. The observed vulnerabilities among females, younger adults, and low-SES groups highlight environmental justice considerations in air quality management. These findings reinforce the role of chronic air pollution exposure as a critical driver of neuropsychiatric disease progression, with profound implications for environmental health policy and equitable mental healthcare.

## Figures and Tables

**Table 1:** The characteristics of the study population by different status during follow-up in the UK Biobank cohort.

Characteristic	Overall	Emotional Disorder Positive	Alzheimer’s Disease	Anxiety Disorder	Stroke	Depression	Epilepsy	Migraine	Parkinson’s Disease	Sleep Disorders
	*n* = 502,356	*n* = 162,160	*n* = 4877	*n* = 11,191	*n* = 6166	*n* = 9652	*n* = 3888	*n* = 3757	*n* = 2453	*n* = 5259
Age (y)	56.5 (8.09)	54.99 (8.11)	69.77 (8.52)	67.74 (8.77)	68.45 (8.78)	66.36 (8.87)	67.14 (8.89)	67.86 (8.80)	68.26 (8.89)	67.14 (8.83)
Sex										
Female	273,294 (54.4)	93,641 (57.7)	2549 (52.3)	5846 (52.2)	3249 (52.7)	5047 (52.3)	2041 (52.5)	1937 (51.6)	1314 (53.6)	2731 (51.9)
Male	229,062 (45.6)	68,519 (42.3)	2328 (47.7)	5345 (47.8)	2917 (47.3)	4605 (47.7)	1847 (47.5)	1820 (48.4)	1139 (46.4)	2528 (48.1)
Townsend deprivation index										
Low	167,330 (33.3)	48,079 (29.6)	1702 (34.9)	3986 (35.6)	2143 (34.8)	3376 (35.0)	1378 (35.4)	1342 (35.7)	873 (35.6)	1839 (35.0)
Moderate	167,150 (33.3)	51,474 (31.7)	1692 (34.7)	3864 (34.5)	2154 (34.9)	3314 (34.3)	1325 (34.1)	1279 (34.0)	854 (34.8)	1811 (34.4)
High	167,240 (33.3)	62,361 (38.5)	1483 (30.4)	3341 (29.9)	1869 (30.3)	2962 (30.7)	1185 (30.5)	1136 (30.2)	726 (29.6)	1609 (30.6)
Education										
Low (<20 years)	341,254 (67.9)	110,765 (68.3)	3009(63.7)	7047(63.0)	3900 (63.3)	6085 (63.0)	2457 (62.2)	2381 (63.4)	1520 (62.0)	3308 (62.9)
High (≥20 years)	161,102 (32.1)	51,395 (31.7)	1772 (36.3)	4144 (37.0)	2266 (36.7)	3567 (37.0)	1431 (36.8)	1376 (36.6)	933 (38.0)	1951 (37.1)
Race										
White	454,124 (90.6)	144,952 (89.4)	4434 (90.9)	10,252 (91.6)	5673 (92.0)	8833 (91.5)	3565 (91.7)	3441 (91.6)	2233 (91.0)	4803 (91.3)
Other	47,332 (9.43)	17,208 (10.6)	443 (9.1)	939 (8.4)	493 (8.0)	819 (8.5)	323 (8.3)	316 (8.4)	220 (9.0)	456 (8.7)
Occupation										
Employment	320,042 (63.8)	114,104 (70.4)	3306 (67.8)	7360 (65.8)	4045 (65.6)	6409 (66.4)	2568 (66.0)	2494 (66.4)	1627 (66.3)	3481 (66.2)
Retired	166,961 (33.3)	42,363 (26.1)	1456 (29.9)	3558 (31.8)	1980 (32.1)	3017 (31.3)	1238 (31.8)	1153 (30.7)	765 (31.2)	1647 (31.3)
Other	15,351 (3.1)	5798 (2.0)	115 (2.4)	273 (2.5)	141 (2.3)	226 (2.4)	82 (2.0)	110 (2.9)	61 (2.5)	131 (2.5)
BMI										
Underweight	2626 (0.53)	1058 (0.7)	26 (0.5)	45 (0.4)	28 (0.5)	39 (0.4)	25 (0.6)	22 (0.6)	13 (0.5)	22 (0.4)
Normal weight	157,365 (31.5)	48,496 (29.9)	1629 (33.4)	3746 (33.5)	2068 (33.5)	3173 (32.9)	1260 (32.4)	1260 (33.5)	815 (33.2)	1740 (33.1)
Overweight	214,154 (42.9)	65,425 (40.3)	2081 (42.7)	4782 (42.7)	2653 (43.0)	4110 (42.6)	1696 (43.6)	1575 (41.9)	1061 (43.3)	2261 (43.0)
Obesity	125,104 (25.1)	46,104 (28.4)	1141 (23.4)	2618 (23.4)	1417 (23.0)	2330 (24.1)	907 (23.3)	900 (24.0)	564 (23.0)	1236 (23.5)
IPAQ										
Low	65,231 (16.5)	26,337 (16.2)	760 (15.6)	1739 (15.5)	963 (15.6)	1566 (16.2)	616 (15.8)	593 (15.8)	391 (15.9)	847 (16.1)
Moderate	155,787 (39.5)	51,501 (31.8)	1914 (39.2)	4389 (39.2)	2427 (39.4)	3749 (38.8)	1551 (39.9)	1465 (39.0)	976 (39.8)	2072 (39.4)
High	173,442 (44.0)	50,846 (31.4)	2203 (45.2)	5063 (45.2)	2776 (45.0)	4337 (44.9)	1721 (44.3)	1699 (45.2)	1086 (44.3)	2340 (44.5)
Smoking status										
Never	273,447 (54.4)	84,181 (51.9)	2720 (55.8)	6250 (55.8)	3454 (56.0)	5403 (56.0)	2163 (55.6)	2119 (56.4)	1382 (56.3)	2952 (56.1)
Previous	173,001 (34.4)	55,074 (34.0)	1688 (34.6)	3869 (34.6)	2113 (34.3)	3294 (34.1)	1380 (35.5)	1287 (34.3)	844 (34.4)	1816 (34.5)
Current	52,958 (10.5)	22,445 (13.8)	465 (9.5)	1045 (9.3)	589 (9.6)	933 (9.7)	336 (8.6)	342 (9.1)	223 (9.1)	479 (9.1)
Alcohol consumption										
Never	22,377 (4.45)	7682 (4.7)	183 (3.8)	388 (3.5)	194 (3.1)	347 (3.6)	124 (3.2)	129 (3.4)	97 (4.0)	185 (3.5)
Previous	18,091 (3.60)	7755 (4.8)	150 (3.1)	392 (3.5)	202 (3.3)	319 (3.3)	130 (3.3)	126 (3.4)	89 (3.6)	178 (3.4)
Current	460,234 (91.6)	146,556 (90.4)	4543 (93.2)	10,409 (93.0)	5767 (93.5)	8983 (93.1)	3633 (93.4)	3501 (93.2)	2267 (92.4)	4895 (93.1)
Sleeplessness										
Rarely	120,735 (24.0)	25,445 (15.7)	1246 (25.5)	2934 (26.2)	1631 (26.5)	2460 (25.5)	1053 (27.1)	992 (26.4)	639 (26.0)	1368 (26.0)
Sometimes	238,758 (47.5)	72,850 (44.9)	2328 (47.7)	5265 (47.0)	2895 (47.0)	4535 (47.0)	1806 (46.5)	1753 (46.7)	1138 (46.4)	2447 (46.5)
Usually	141,358 (28.1)	63,779 (39.3)	1303 (26.7)	2987 (26.7)	1637 (26.5)	2653 (27.5)	1028 (26.4)	1011 (26.9)	676 (27.6)	1443 (27.4)
Family history of Parkinson’s disease										
Yes	56,356 (11.2)	17,717 (10.9)	549 (11.3)	1138 (10.2)	670 (10.9)	987 (10.2)	453 (11.7)	393 (10.5)	271 (11.0)	583 (11.1)
No	445,987 (88.8)	144,443 (89.1)	4328 (88.7)	10,053 (89.8)	5496 (89.1)	8665 (89.8)	3435 (88.3)	3364 (89.5)	2182 (89.0)	4676 (88.9)
Family history of Alzheimer’s disease										
Yes	39,574 (7.9)	12,548 (7.7)	438 (9.0)	989 (8.8)	557 (9.0)	872 (9.0)	333 (8.6)	323 (8.6)	225 (9.2)	472 (9.0)
No	462,769 (92.1)	149,612 (92.3)	4439 (91.0)	10,202 (91.2)	5609 (91.0)	8780 (91.0)	3555 (91.4)	3434 (91.4)	2228 (90.8)	4787 (91.0)
Family history of depression										
Yes	23,402 (4.7)	10,252 (6.3)	239 (4.9)	570 (5.1)	341 (5.5)	489 (5.1)	187 (4.8)	191 (5.1)	133 (5.4)	283 (5.4)
No	478,941 (95.3)	151,908 (93.7)	4638 (95.1)	10,621 (94.9)	5825 (94.5)	9163 (94.9)	3701 (95.2)	3566 (94.9)	2320 (94.6)	4976 (94.6)

Note: TDI denotes Townsend deprivation index. BMI denotes the Body Mass Index. IPAQ denotes the International Physical Activity Questionnaire.

**Table 2:** Hazard ratios of per interquartile range increases in air pollutants on two transition stages of psychiatric and neurological disorders during follow-up.

Disease	PM_2.5_		NO_x_	
	Stage 2	Stage 3	Stage 2	Stage 3
Alzheimer’s disease	**1.13 (1.06–1.21)**	**1.20 (1.09–1.32)**	**1.05 (1.00–1.11)**	1.05 (0.98–1.14)
Anxiety	**1.10 (1.06–1.14)**	**1.16 (1.09–1.22)**	**1.05 (1.02–1.09)**	**1.07 (1.02–1.13)**
Stroke	**1.11 (1.05–1.17)**	**1.17 (1.08–1.27)**	**1.05 (1.01–1.10)**	1.06 (0.99–1.13)
Depression	**1.10 (1.05–1.14)**	**1.17 (1.10–1.24)**	**1.05 (1.02–1.09)**	**1.07 (1.01–1.13)**
Epilepsy	**1.08 (1.02–1.15)**	**1.14 (1.03–1.25)**	1.05 (0.99–1.10)	1.06 (0.97–1.15)
Migraine	**1.10 (1.03–1.17)**	**1.17 (1.06–1.28)**	1.04 (0.99–1.10)	**1.10 (1.01–1.19)**
Parkinson’s disease	**1.10 (1.02–1.19)**	**1.15 (1.02–1.29)**	1.05 (0.98–1.12)	1.08 (0.98–1.20)
Sleep disorders	**1.09 (1.03–1.15)**	**1.14 (1.05–1.24)**	**1.05 (1.00–1.10)**	1.06 (0.98–1.13)

Note: The interquartile range of PM_2.5_ and NO_x_ were 3.27 μg/m^3^ and 9.07 μg/m^3^. In stage 1 (defined as from the baseline to the diagnosis of mood disorders but before the onset of neuropsychiatric disorders), per IQR changes in PM_2.5_ and NO_x_ were associated with a hazard ratio of 1.28 (95% CI: 1.27, 1.30) and 1.10 (95% CI: 1.09, 1.11), respectively. Stage 2 was defined as the disease progression from baseline to an ICD-10 clinical diagnosis and stage 3 as from a PHQ-4-positive status to a subsequent clinical diagnosis. The bolded estimates denote statistically significant values.

## Data Availability

This study was conducted using data from the UK Biobank, which maintains a controlled access policy requiring researcher registration and approval. (Application Number 95736; http://www.ukbiobank.ac.uk/ (accessed on 25 October 2023).

## Supplementary Materials

The following supporting information can be downloaded at https://www.mdpi.com/article/10.3390/toxics14010004/s1. Table S1. UKB data fields with corresponding variables and assigned values. Table S2 Descriptive table of pollutant concentrations (μg/m³). Table S3 Associations of IQR increases in Air Pollutants with stage 1. Table S4 Sensitivity analysis 1: Incorporation of additional covariates (depression, Parkinson’s syndrome and family history of diseases). Table S5 Sensitivity Analysis 2: Exclusion of individuals developed mental health disorders within the first-year post-enrollment. Table S6 Sensitivity Analysis 3: Multiple imputation using chain equations for missing data in key covariates.

## References

[1] (2024). Global burden of 288 causes of death and life expectancy decomposition in 204 countries and territories and 811 subna-tional locations, 1990-2021: A systematic analysis for the Global Burden of Disease Study 2021. Lancet.

[2] Chen Q., Huang S., Xu H., Peng J., Wang P., Li S., Zhao J., Shi X., Zhang W., Shi L. (2024). The burden of mental disorders in Asian countries, 1990–2019: An analysis for the global burden of disease study 2019. Transl. Psychiatry.

[3] Sisodiya S.M., I Gulcebi M., Fortunato F., Mills J.D., Haynes E., Bramon E., Chadwick P., Ciccarelli O., David A.S., De Meyer K. (2024). Climate change and disorders of the nervous system. Lancet Neurol..

[4] GBD 2019 Mental Disorders Collaborators (2022). Global, regional, and national burden of 12 mental disorders in 204 countries and territories, 1990–2019: A systematic analysis for the Global Burden of Disease Study 2019. Lancet Psychiatry.

[5] GBD 2016 Neurology Collaborators (2019). Global, regional, and national burden of neurological disorders, 1990-2016: A systematic analysis for the Global Burden of Disease Study 2016. Lancet Neurol..

[6] GBD 2019 Dementia Forecasting Collaborators (2022). Estimation of the global prevalence of dementia in 2019 and forecasted prevalence in 2050: An analysis for the Global Burden of Disease Study 2019. Lancet Public Health.

[7] Landrigan P.J., Fuller R., Acosta N.J.R., Adeyi O., Arnold R., Basu N., Baldé A.B., Bertollini R., Bose-O’Reilly S., Boufford J.I. (2018). The Lancet Commission on pollution and health. Lancet.

[8] Hahad O., Lelieveld J., Birklein F., Lieb K., Daiber A., Münzel T. (2020). Ambient Air Pollution Increases the Risk of Cerebrovascular and Neuropsychiatric Disorders through Induction of Inflammation and Oxidative Stress. Int. J. Mol. Sci..

[9] Zhang Y., Xu J., Shi J., Ma Y., Yu N., Zhou X., Li X., Wang T., Jia G., Chen Z. (2025). PM2.5 and O_3_ co-exposure affecting serum LDL-C: Evidence from ep-idemiology and animal models. J. Environ. Expo. Assess..

[10] Wu Y., Zhang Y., Wang J., Gan Q., Su X., Zhang S., Ding Y., Yang X., Zhang N., Wu K. (2025). Genetic evidence for the causal effects of air pollution on the risk of respiratory diseases. Ecotoxicol. Environ. Saf..

[11] Song J., Han K., Wang Y., Qu R., Liu Y., Wang S., Wang Y., An Z., Li J., Wu H. (2022). Microglial Activation and Oxidative Stress in PM_2.5_-Induced Neurodegenerative Disorders. Antioxidants.

[12] Shi L., Zhu Q., Wang Y., Hao H., Zhang H., Schwartz J., Amini H., van Donkelaar A., Martin R.V., Steenland K. (2023). Incident dementia and long-term exposure to constituents of fine particle air pollution: A national cohort study in the United States. Proc. Natl. Acad. Sci. USA.

[13] Fan H., Li J., Dou Y., Yan Y., Wang M., Yang X., Ma X. (2024). Linking ambient air pollution to mental health: Evidence based on the two-sample Mendelian randomization and colocalization study. Transl. Psychiatry.

[14] Borroni E., Buoli M., Nosari G., Ceresa A., Fedrizzi L., Antonangeli L.M., Monti P., Bollati V., Pesatori A.C., Carugno M. (2024). Impact of air pollution exposure on the severity of major depressive disorder: Results from the DeprAir study. Eur. Psychiatry.

[15] Grande G., Ljungman P.L.S., Eneroth K., Bellander T., Rizzuto D. (2020). Association Between Cardiovascular Disease and Long-term Exposure to Air Pollution With the Risk of Dementia. JAMA Neurol..

[16] Atkinson R.W., Carey I.M., Kent A.J., van Staa T.P., Anderson H.R., Cook D.G. (2013). Long-term exposure to outdoor air pollution and incidence of cardiovascular diseases. Epidemiology.

[17] Zabihi S., Bestwick J.P., Jitlal M., Bothongo P.L., Zhang Q., Carter C., Roche M., Morgan-Trimmer S., Birks Y., Wilberforce M. (2025). Early presentations of dementia in a diverse population. Alzheimer’s Dement..

[18] Benasi G., Fava G.A., Guidi J. (2021). Prodromal Symptoms in Depression: A Systematic Review. Psychother. Psychosom..

[19] Lång U., Ramsay H., Yates K., Veijola J., Gyllenberg D., Clarke M.C., Leacy F.P., Gissler M., Kelleher I. (2022). Potential for prediction of psychosis and bipolar disorder in Child and Adolescent Mental Health Services: A longitudinal register study of all people born in Finland in 1987. World Psychiatry.

[20] Deng W., Chong B., Addington J., Bearden C.E., Cadenhead K.S., Cornblatt B.A., Keshavan M., Mathalon D.H., Perkins D.O., Stone W. (2025). Beyond the Descriptive: A Comprehensive, Multidomain Validation of Symptom Trajectories for Individuals at Clinical High Risk for Psychosis. Biol. Psychiatry Cogn. Neurosci. Neuroimaging.

[21] Sudlow C., Gallacher J., Allen N., Beral V., Burton P., Danesh J., Downey P., Elliott P., Green J., Landray M. (2015). UK biobank: An open access resource for identifying the causes of a wide range of complex diseases of middle and old age. PLoS Med..

[22] Gao X., Jiang M., Huang N., Guo X., Huang T. (2023). Long-Term Air Pollution, Genetic Susceptibility, and the Risk of Depression and Anxiety: A Prospective Study in the UK Biobank Cohort. Environ. Health Perspect..

[23] Robison L.S., Gannon O.J., Thomas M.A., Salinero A.E., Abi-Ghanem C., Poitelon Y., Belin S., Zuloaga K.L. (2020). Role of sex and high-fat diet in metabolic and hypothalamic disturbances in the 3xTg-AD mouse model of Alzheimer’s disease. J. Neuroinflammation.

[24] Pugsley K.L., Stedman J.R., Brookes D.M., Kent A.J., Morris R.J., Whiting S.L. Technical report on UK supplementary modelling assessment under the Air Quality Standards Regulations 2010 for 2020. Report for The Department for Environment, Food and Rural Affairs, the Welsh Government, the Scottish Government and the Department of the Environment for Northern Ireland.

[25] Santana J., Miranda A., Yamamura C., Filho S., Tambourgi E., Ho L., Berssaneti F. (2020). Effects of air pollution on human health and costs: Current situation in São Paulo, Brazil. Sustainability.

[26] Li W.Q., Yang L., Wang S.F., Zhang L.W., Sheng C., Huang Y.B. (2021). Application of multi-stage competing risk model to survival data. Chin. J. Prev. Med..

[27] de Wreede L.C., Fiocco M., Putter H. (2010). The mstate package for estimation and prediction in non- and semi-parametric multi-state and competing risks models. Comput. Methods Programs Biomed..

[28] Liu J., Zuo S.W., Li Y., Jia X., Jia S.H., Zhang T., Song Y.X., Wei Y.Q., Xiong J., Hu Y.H. (2016). Hyperhomocysteinaemia is an independent risk factor of abdominal aortic aneurysm in a Chinese Han population. Sci. Rep..

[29] Yang S., Li M., Guo C., Requia W.J., Sakhvidi M.J.Z., Lin K., Zhu Q., Chen Z., Cao P., Yang L. (2025). Associations of long-term exposure to nitrogen oxides with all-cause and cause-specific mortality. Nat. Commun..

[30] Braithwaite I., Zhang S., Kirkbride J.B., Osborn D.P.J., Hayes J.F. (2019). Air Pollution (Particulate Matter) Exposure and Associations with Depression, Anxiety, Bipolar, Psychosis and Suicide Risk: A Systematic Review and Meta-Analysis. Environ. Health Perspect..

[31] Fu P., Yung K.K.L. (2020). Air Pollution and Alzheimer’s Disease: A Systematic Review and Meta-Analysis. J. Alzheimer’s Dis..

[32] Qiu X., Shi L., Kubzansky L.D., Wei Y., Castro E., Li H., Weisskopf M.G., Schwartz J.D. (2023). Association of Long-term Exposure to Air Pollution With Late-Life Depression in Older Adults in the US. JAMA Netw. Open.

[33] Yang B.-Y., Guo Y., Markevych I., Qian Z., Bloom M.S., Heinrich J., Dharmage S.C., Rolling C.A., Jordan S.S., Komppula M. (2019). Association of Long-term Exposure to Ambient Air Pollutants With Risk Factors for Cardiovascular Disease in China. JAMA Netw. Open.

[34] Abed Al Ahad M., Demšar U., Sullivan F., Kulu H. (2023). Long-term exposure to air pollution and mortality in Scotland: A reg-ister-based individual-level longitudinal study. Environ. Res..

[35] Ma K.J., Lin Y.J., Liu C.S., Tseng P.Y., Wang S.H., Yao C.Y., Wang J.Y. (2023). Association between 14 candidate genes, PM2.5, and af-fective disorders: A study of the Taiwan Biobank. BMC Public Health.

[36] Monfared A.A.T., Fu S., Hummel N., Qi L., Chandak A., Zhang R., Zhang Q. (2023). Estimating Transition Probabilities Across the Alzheimer’s Disease Continuum Using a Nationally Representative Real-World Database in the United States. Neurol. Ther..

[37] Wu Y., Zhang S., Qian S.E., Cai M., Li H., Wang C., Zou H., Chen L., Vaughn M.G., McMillin S.E. (2022). Ambient air pollution associated with incidence and dynamic progression of type 2 diabetes: A trajectory analysis of a population-based cohort. BMC Med..

[38] Luo H., Zhang Q., Yu K., Meng X., Kan H., Chen R. (2022). Long-term exposure to ambient air pollution is a risk factor for trajectory of cardiometabolic multimorbidity: A prospective study in the UK Biobank. EBioMedicine.

[39] Lee Y., Lee D., Jung H., Cho Y., Baek J.H., Hong K.S. (2022). Heterogeneous early illness courses of Korean patients with bipolar disorders: Replication of the staging model. BMC Psychiatry.

[40] Liu D., Zhao Y., Qi Y., Gao Y., Tu D., Wang Y., Gao H.-M., Zhou H. (2020). Benzo(a)pyrene exposure induced neuronal loss, plaque deposition, and cognitive decline in APP/PS1 mice. J. Neuroinflamm..

[41] Zhu L.-J., Li F., Zhu D.-Y. (2023). nNOS and Neurological, Neuropsychiatric Disorders: A 20-Year Story. Neurosci. Bull..

[42] Ye S., Li S., Ma Y., Wei L., Zeng Y., Hu D., Xiao F. (2022). Ambient NO2 exposure induces migraine in rats: Evidence, mechanisms and interventions. Sci. Total. Environ..

[43] Cía A.H., Stagnaro J.C., Gaxiola S.A., Vommaro H., Loera G., Medina-Mora M.E., Sustas S., Benjet C., Kessler R.C. (2018). Lifetime prevalence and age-of-onset of mental disorders in adults from the Argentinean Study of Mental Health Epidemiology. Soc. Psychiatry Psychiatr. Epidemiol..

[44] Marcus S.M., Young E.A., Kerber K.B., Kornstein S., Farabaugh A.H., Mitchell J., Wisniewski S.R., Balasubramani G., Trivedi M.H., Rush A.J. (2005). Gender differences in depression: Findings from the STAR*D study. J. Affect. Disord..

[45] Silveira P.P., Pokhvisneva I., Howard D.M., Meaney M.J. (2023). A sex-specific genome-wide association study of depression phenotypes in UK Biobank. Mol. Psychiatry.

[46] Luppa M., Sikorski C., Luck T., Ehreke L., Konnopka A., Wiese B., Weyerer S., König H.-H., Riedel-Heller S. (2012). Age- and gender-specific prevalence of depression in latest-life—Systematic review and meta-analysis. J. Affect. Disord..

[47] Jacquemont S., Coe B.P., Hersch M., Duyzend M.H., Krumm N., Bergmann S., Beckmann J.S., Rosenfeld J.A., Eichler E.E. (2014). A higher mutational burden in females supports a “female protective model” in neurodevelopmental disorders. Am. J. Hum. Genet..

[48] Deng Y., Sun S., Wu S., Chen K., Liu Y., Wei W., Bei N., Qiu C., Li X. (2024). Burden and trends of mental disorders in China from 1990 to 2019: Findings from the Global Burden of Disease Study 2019. Soc. Psychiatry Psychiatr. Epidemiol..

[49] Solmi M., Radua J., Olivola M., Croce E., Soardo L., de Pablo G.S., Shin J.I., Kirkbride J.B., Jones P., Kim J.H. (2022). Age at onset of mental disorders worldwide: Large-scale meta-analysis of 192 epidemiological studies. Mol. Psychiatry.

[50] Ni Y., Zhou Y., Kivimäki M., Cai Y., Carrillo-Larco R.M., Xu X., Dai X., Xu X. (2023). Socioeconomic inequalities in physical, psychological, and cognitive multimorbidity in middle-aged and older adults in 33 countries: A cross-sectional study. Lancet Healthy Longev..

[51] Li N., Chen S., Wu Z., Dong J., Wang J., Lei Y., Mo J., Wei W., Li T. (2024). Secular trends in the prevalence of schizophrenia among different age, period and cohort groups between 1990 and 2019. Asian J. Psychiatry.

[52] Cho M.J., Chang S.M., Hahm B.-J., Chung I.-W., Bae A., Lee Y.M., Ahn J.H., Won S., Son J., Hong J.P. (2012). Lifetime risk and age of onset distributions of psychiatric disorders: Analysis of national sample survey in South Korea. Soc. Psychiatry Psychiatr. Epidemiol..

[53] Pols H. (2007). August Hollingshead and Frederick Redlich: Poverty, Socioeconomic Status, and Mental Illness. Am. J. Public Health.

[54] Zhou Y., Kivimäki M., Yan L.L., Carrillo-Larco R.M., Zhang Y., Cheng Y., Wang H., Zhou M., Xu X. (2024). Associations between socioeconomic ine-qualities and progression to psychological and cognitive multimorbidities after onset of a physical condition: A multi-cohort study. EClinicalMedicine.

[55] Wang D., Hou Y., Wang Y., Zhao X., Ding Y. (2025). Behavioral variations and their effects on traffic-related PM_2.5_ inhalation exposure: Findings from three cross-sectional surveys (2015–2020). J. Environ. Expo. Assess..

[56] Smith M.L., Steinman L.E., Montoya C.N., Thompson M., Zhong L., Merianos A.L. (2023). Effectiveness of the Program to Encourage Active, Rewarding Lives (PEARLS) to reduce depression: A multi-state evaluation. Front. Public Health.

