# Supplementary material for: Long-Term Urban Air Pollution Drives Multi-Stage Neuropsychiatric Disorder Trajectories: A Prospective Cohort Study
Source: Toxics. 2025 Dec 19;14(1):4. doi: 10.3390/toxics14010004 (PMC12845905; doi:10.3390/toxics14010004)
Supplement: Supplementary file 1 [file toxics-14-00004-s001.zip › toxics-4021570-supplementary.pdf]

Supporting Information

Long-term Urban Air Pollution Drives Multistage Neuropsychiatric Disorder Trajectories: A Prospective Cohort Study

Yuanyuan Song[1, †], Shiqing Zhang[2,3,4, †], Siru Yang[1], Xiaoke Gao[1], Lei Shi[2,3,4],Jinjian Chen[1], Kaili Lin[1,*], Jun Yang[1, *]

[1] School of Public Health, Guangzhou Medical University, Guangzhou, 511436, China

[2] State Key Laboratory of Bioactive Molecules and Druggability Assessment, Guangdong Basic Research Center of Excellence for Natural Bioactive Molecules and Discovery of Innovative Drugs, Jinan University, Guangzhou 510632, China

[3] JNU-HKUST Joint Laboratory for Neuroscience and Innovative Drug Research, College of Pharmacy, Jinan University, Guangzhou 510632, China

[4] Guangdong Province Key Laboratory of Pharmacodymamic Constituents of TCM & New Drugs Research, Guangdong Hong Kong-Macau Joint Laboratory for Pharmacodynamic Constituents of TCM and New Drugs Research, Jinan University, Guangzhou 510632, China

[†] These authors contributed equally: Yuanyuan Song, Shiqing Zhang

* Corresponding authors: Kaili Lin, lin_kaili@gzhmu.edu.cn; Jun Yang, yangjun@gzhmu.edu.cn.

Contents

Table S1 UKB data fields with corresponding variables and assigned values.

Table S2 Descriptive Table of Pollutant Concentrations (μg/m³).

Table S3 Associations of IQR increases in Air Pollutants with stage 1.

Table S4 Sensitivity Analysis 1: Incorporation of additional covariates (smoking, alcohol intake, insomnia, and family history of diseases).

Table S5 Sensitivity Analysis 2: Exclusion of individuals developed mental health disorders within the first-year post-enrollment.

Table S6 Sensitivity Analysis 3: Multiple imputation using chain equations for missing data in key covariates.

Table S1 UKB data fields with corresponding variables and assigned values.

Variable	UK Biobank code	Value
Age	21022-0.0	-
TDI	189-0.0	1= high,<br>2= medium,<br>3= low
Sex	31-0.0	0= female,<br>1= male
Educational level	6138-0.0	Low：≤20 years<br>High：≥20 years
Ethnic background	21000-0.0	0= Caucasian,<br>1= non-Caucasian
Current employment status	6142-0.0	1= active,<br>2= retired,<br>3= other status
Body Mass Index	21001-0.0	<18.5= underweight,<br>18.5-24.9= normal,<br>25.0-29.9= overweight,<br>≥30.0= obese
Physical Activity Assessment	22032-0.0	0= mild,<br>1= moderate,<br>2= high intensity,<br>3= deletion
Smoking status	20116-0.0	1=Rarely,<br>2=Some time<br>3=Usually
Drinking status	20117-0.0	1=Rarely,<br>2=Some time<br>3=Usually
Sleeplessness	1200-0.0	1=Rarely,<br>2=Some time<br>3=Usually
Family history of depression	20107-0.0，20110-0.0，20111-0.0	0= no, 1= yes
Family history of Parkinson's syndrome	20107-0.0，20110-0.0，20111-0.0	0= no, 1= yes
Family history of Alzheimer's disease	20107-0.0，20110-0.0，20111-0.0	0= no, 1= yes

Table S2 Descriptive table of pollutant concentrations (μg/m³).

Variable	Min.	1st	Median	Mean	3rd	Max.
$PM_{2.5}$	0.28	8.27	9.92	9.99	11.55	23.98
NO_x	0.38	13.11	17.57	18.19	22.19	65.21

Table S3 Associations of IQR increases in Air Pollutants with stage 1.

Mental disorders (stage 1)	$PM_{2.5}$		NOx	
	**HR (95%CI)**	**P-value**	**HR (95%CI)**	**P-value**
**Main model**	1.28 (1.27–1.30)		1.10 (1.09–1.11)	
**Age**				
≥60	1.07 (1.066-1.079)	0.382	1.01 (1.008-1.011)	0.330
<60	1.08 (1.076-1.084)		1.01 (1.009-1.012)	
**Sex**				
Female	1.08(1.075-1.085)	0.379	1.01 (1.009-1.012)	0.222
Male	1.08(1.072-1.083)		1.01 (1.009-1.012)	
**TID**				
High	1.049(1.042-1.056)	<0.001	0.998(0.996-1.000)	<0.001
Moderate	1.069(1.062-1.076)		1.008(1.006-1.010)	
Low	1.082(1.077-1.087)		1.011(1.009-1.012)	

Table S4 Sensitivity analysis 1: Incorporation of additional covariates (depression, Parkinson's syndrome and family history of diseases).

**Diseases**	**$PM_{2.5}$**	**NO_x**
**Alzheimer's disease**		
Stage 2	**1.13(1.06–1.21)**	**1.05(1.00–1.11)**
Stage 3	**1.20(1.09–1.32)**	1.05(0.98–1.13)
**Anxiety**		
Stage 2	**1.10(1.06–1.14)**	**1.05(1.02–1.09)**
Stage 3	**1.16(1.09–1.22)**	**1.07(1.02–1.13)**
**Stroke**		
Stage 2	**1.11(1.05–1.17)**	**1.05(1.01–1.10)**
Stage 3	**1.17(1.08–1.27)**	1.06(0.99–1.13)
**Depression**		
Stage 2	**1.10(1.05–1.14)**	**1.05(1.02–1.09)**
Stage 3	**1.17(1.09–1.24)**	**1.07(1.01–1.12)**
**Epilepsy**		
Stage 2	**1.08(1.02–1.15)**	**1.05(1.00–1.10)**
Stage 3	**1.14(1.03–1.25)**	1.06(0.97–1.15)
**Migraine**		
Stage 2	**1.10(1.03–1.17)**	1.04(0.99–1.10)
Stage 3	**1.17(1.06–1.28)**	**1.10(1.01–1.19)**
**Parkinson's disease**		
Stage 2	**1.10(1.02–1.19)**	1.05(0.98–1.12)
Stage 3	**1.15(1.02–1.29)**	1.08(0.98–1.20)
**Sleep disorders**		
Stage 2	**1.08(1.03–1.15)**	**1.05(1.00–1.10)**
Stage 3	**1.14(1.05–1.24)**	1.06(0.98–1.13)

Note: The HRs of $PM_{2.5}$ and NO_x were 1.28 (1.27–1.30) and 1.10 (1.09–1.11) at the Stage 1.

Table S5 Sensitivity Analysis 2: Exclusion of individuals developed mental health disorders within the first-year post-enrollment.

Diseases	$PM_{2.5}$	NO_x
**Alzheimer's disease**		
Stage 2	**1.13(1.06–1.21)**	**1.05(1.00–1.11)**
Stage 3	**1.20(1.09–1.32)**	1.05(0.98–1.13)
**Anxiety**		
Stage 2	**1.10(1.06–1.14)**	**1.06(1.02–1.09)**
Stage 3	**1.16(1.09–1.22)**	**1.07(1.02–1.13)**
**Stroke**		
Stage 2	**1.10(1.04–1.16)**	**1.05(1.00–1.10)**
Stage 3	**1.17(1.08–1.27)**	1.06(0.99–1.13)
**Depression**		
Stage 2	**1.09(1.05–1.14)**	**1.05(1.01–1.09)**
Stage 3	**1.17(1.10–1.24)**	**1.07(1.01–1.12)**
**Epilepsy**		
Stage 2	**1.07(1.00–1.14)**	1.04(0.99–1.10)
Stage 3	**1.14(1.03–1.25)**	1.06(0.97–1.15)
**Migraine**		
Stage 2	**1.10(1.03–1.18)**	1.05(0.99–1.11)
Stage 3	**1.17(1.06–1.28)**	**1.10(1.01–1.19)**
**Parkinson's disease**		
Stage 2	**1.10(1.02–1.20)**	1.04(0.98–1.12)
Stage 3	**1.15(1.02–1.29)**	1.08(0.98–1.20)
**Sleep disorders**		
Stage 2	**1.09(1.03–1.15)**	**1.05(1.00–1.10)**
Stage 3	**1.14(1.05–1.24)**	1.06(0.98–1.13)

Note: The HRs of $PM_{2.5}$ and NO_x were 1.28(1.27–1.30) and 1.10(1.09–1.11) at the Stage 1.

Table S6 Sensitivity Analysis 3: Multiple imputation using chain equations for missing data in key covariates.

**Diseases**	**$PM_{2.5}$**	**NO_x**
**Alzheimer's disease**		
Stage 2	**1.13(1.06-1.20)**	**1.05(1.00-1.10)**
Stage 3	**1.20(1.09-1.32)**	1.05(0.98-1.14)
**Anxiety**		
Stage 2	**1.10(1.06-1.14)**	**1.05(1.02-1.09)**
Stage 3	**1.16(1.09-1.22)**	**1.07(1.02-1.13)**
**Stroke**		
Stage 2	**1.11(1.05-1.16)**	**1.05(1.01-1.10)**
Stage 3	**1.17(1.08-1.27)**	1.06(0.99-1.13)
**Depression**		
Stage 2	**1.10(1.06-1.14)**	**1.05(1.02-1.09)**
Stage 3	**1.17(1.10-1.24)**	**1.07(1.01-1.13)**
**Epilepsy**		
Stage 2	**1.08(1.02-1.15)**	**1.05(1.00-1.10)**
Stage 3	**1.14(1.03-1.25)**	1.06(0.97-1.15)
**Migraine**		
Stage 2	**1.10(1.03-1.17)**	1.04(0.99-1.09)
Stage 3	**1.17(1.06-1.28)**	**1.10(1.01-1.19)**
**Parkinson's disease**		
Stage 2	**1.10(1.02-1.19)**	1.05(0.99-1.12)
Stage 3	**1.15(1.02-1.29)**	1.08(0.98-1.20)
**Sleep disorders**		
Stage 2	**1.09(1.03-1.15)**	**1.05(1.01-1.10)**
Stage 3	**1.14(1.05-1.24)**	1.06(0.98-1.13)

Note: The HRs of $PM_{2.5}$ and NO_x were 1.28(1.27–1.30) and 1.10(1.09–1.11) at the Stage 1.
